# A social-ecological trap theory-informed investigation of dietary patterns in southwestern Madagascar

**DOI:** 10.3389/fnut.2026.1745233

**Published:** 2026-02-17

**Authors:** Heather Kelahan, Stephanie M. Wu, Hervet Randriamady, Aroniaina Falinirina, Madeleine Rasoanirina, Frédéric Déclerque, Marc Y. Solofoarimanana, Jean C. Mahefa, Eric B. Rimm, Jessica Fanzo, Aaron C. Hartmann, Emma Gibbons, Gildas Todinanahary, Sebastien Haneuse, Christopher D. Golden

**Affiliations:** 1 Department of Nutrition, Harvard T.H. Chan School of Public Health, Boston, MA, United States; 2 Division of Psychiatry, University College London, London, United Kingdom; 3 Institut Halieutiques et des Sciences Marines, University of Toliara, Toliara, Madagascar; 4 Department of Epidemiology, Harvard T.H. Chan School of Public Health, Boston, MA, United States; 5 The Columbia Climate School, Columbia University, New York, NY, United States; 6 Department of Organismic and Evolutionary Biology, Harvard University, Cambridge, MA, United States; 7 Perry Institute for Marine Science, Waitsfield, VT, United States; 8 Reef Doctor, Ifaty, Madagascar; 9 Department of Biostatistics, Harvard T.H. Chan School of Public Health, Boston, MA, United States

**Keywords:** diet, dietary patterns, food system, Madagascar, social-ecological traps

## Abstract

**Background:**

Social-ecological trap theory highlights the potential for food systems and the social and environmental contexts within which they are situated to ‘trap’ individuals into a trajectory of specific nutritional outcomes when their physical or financial access to traditional foods is restricted, resulting in less healthy dietary patterns than those traditionally consumed.

**Objective:**

While social-ecological trap theory literature highlights the potential for these traps to result in four hypothesized dietary patterns, the presence and composition of such dietary patterns have not been explored in southwestern Madagascar.

**Methods:**

This study employs innovative Weighted Overfit Latent Class Analysis methods to identify dietary patterns among individuals residing in southwestern Madagascar. The study used longitudinal cohort data collected from 2023 to 2024.

**Results:**

Four dietary patterns were identified and characterized as (1) a traditional dietary pattern which included 35.9% (SD 11.1%) of the population, (2) a industrialized-transitioning dietary pattern which included 29.2% (SD 13.5%) of the population, (3) an traditional-undernourishing dietary pattern which included 16.3% (SD 5.2%) of the population, and (4) a industrialized-undernourishing dietary pattern which included 17.8% (SD 11.2%) of the population. The four dietary patterns identified aligned with three of the four patterns hypothesized to result from social-ecological traps. Those in the traditional dietary pattern consumed the most diverse diet and tended to be fishers who also often participated in crop-based agriculture. Those in the industrialized-transitioning dietary pattern consumed a greater proportion of their diet from market-source foods. Those in the traditional-undernourishing dietary pattern consumed the fewest calories and had the lowest level of food security. Lastly, those in the industrialized-undernourishing dietary pattern consumed 63% of their calories from rice and consumed more market-source foods than those in the traditional-undernourishing dietary pattern.

**Conclusion:**

Of the four dietary patterns identified in southwestern Madagascar, two are characterized as higher-quality and two as undernourishing dietary patterns. Each dietary pattern comprises individuals of varying demographic and socio-economic status. Understanding dietary patterns and who follows them enables policymakers and public health practitioners to better understand who may be most affected by the impacts of social and ecological change on the food system, thereby improving the targeting of nutritional interventions.

## Introduction

Interrelated shifts in dietary intake and ecological systems, which form the foundation of the food system, have occurred globally over the past three decades, including in Madagascar (1), a country with a population of over 32 million people. Globally, dietary intake has become increasingly Western and industrialized, characterized by an increased consumption of highly processed, often sugar-sweetened, and calorically dense foods (2), which has led to a higher prevalence of non-communicable diseases, such as heart disease, cancer, chronic respiratory disease, and diabetes (3). While Madagascar experiences lower levels of non-communicable diseases compared to most countries in Sub-Saharan Africa, the nutrition transition has led to an increased prevalence of these outcomes, alongside consistently high rates of undernutrition (4). Madagascar is also one of the most chronically undernourished countries, with a stunting (chronic undernutrition) prevalence among those under five of nearly 40% (10th highest), and one in five women is classified as underweight (5). Micronutrient deficiencies are also highly prevalent with 67% of the population estimated to be deficient in zinc, 15% depleted in vitamin B12, 12% deficient in retinol (6), and almost 40% of reproductive-aged women (age 15–49) and nearly half of children 6–59 months old classified as anemic (4).

Parallel shifts in environmental systems occurred at both the global and local levels. Overall, Madagascar is highly vulnerable to droughts (7) and other extreme weather events, such as cyclones (8, 9), which are predicted to intensify due to climate change (7, 10).

Despite the interconnections between ecological systems, food systems, and nutritional outcomes, environmental changes and nutritional outcomes are often studied and addressed in isolation (11). Treating these shifts as distinct is ineffective, given the numerous links between them (1, 12–15), including the impact of environmental events on food chain stability, prices, and infrastructure (12), factors that ultimately impact the physical availability and affordability of foods. Undesirable outcomes, such as the inability to physically access or afford foods, that result from feedback loops in ecological or socioeconomic systems have been described as social-ecological traps (11). Social-ecological traps are often difficult to ‘escape’ as the environmental and social factors compound on each other, creating pressures that make changing behavior, and thus escaping the trap, difficult. Past research has demonstrated the potential for social-ecological traps to result in specific nutritional outcomes in the context of coral reef-based food systems (11), such as those found in southwestern Madagascar. Four potential dietary patterns were hypothesized, including a *traditional* dietary pattern, enabled by continued access to diverse and nutrient dense traditional foods, a *mixed* dietary pattern brought about by continued access to traditional foods alongside increased access to more processed market-source foods, an *overnourishing* dietary pattern caused by reduced access to traditional source foods and increased reliance on processed market-source foods, and lastly an *undernourishing* dietary pattern caused by reduced access to traditional source foods with no availability of replacement market-source foods (11).

In this study, we examine the presence and composition of dietary patterns in a coral reef-based food system in southwestern Madagascar, how they compare to the dietary patterns previously hypothesized to result from social-ecological traps in coral reef-based food systems, the prevalence of each dietary pattern, and the demographics of those who are part of them.

## Methods

### Population

The data for this study come from the Health Impacts of Artificial Reef Advancement (HIARA) longitudinal study cohort, established in 2023 in southwestern Madagascar (16). The HIARA cohort is part of the larger ARMS Restore project that aims to rebuild coral reefs in Madagascar to restore biodiversity, build fisheries, and improve human health. A census of 91% of households in the region served as the sampling frame, from which a stratified random sample of 462 households was selected (16). This analysis aims to understand the potential for social-ecological traps in reef-based food systems, and therefore, only a subsample of households from the main study was selected. Specifically, the 385 households from the 12 coastal villages were included (representing 1,760 individuals). Individuals from selected households participated in up to eight waves of quarterly socio-economic, health, and dietary intake surveys, conducted on: (1) April/May 2023, (2) June/July 2023, (3) October/November 2023, (4) January/February 2024, (5) April/May 2024, (6) June/July 2024, (7) October/November 2024, and (8) January/February 2025. To minimize the impact of day-to-day variation in dietary intake on the measure of average dietary intake for each individual, this analysis included the 96.6% of households who completed more than one round of data collection (372 households, representing 1,702 individuals). Two or more rounds of dietary intake data help to reduce the impact of day-to-day variation (17). The cutoff of two or more rounds of dietary intake data was selected to balance reducing day-to-day variation in diet with maintaining an adequate sample size. The average number of completed rounds of data collection for those included in the study was 7.1.

### Ethics approval and consent to participate

Recruitment, enrollment, and collection of verbal consent to participate were conducted in accordance with our IRB-approved study (Protocol #20–1944 and 22–0491, Committee on the Use of Human Subjects, Office of Human Research Administration at the Harvard T. H. Chan School of Public Health). The study was also approved by the Ethics Committee of the Malagasy Ministry of Public Health (N036MSANP/SG/AMM/CERBM) and the local medical inspector in Toliara (16).

### Measures

#### Method of assessing dietary intake

This study used longitudinal data to estimate long-term dietary intake for each individual in the study. As measuring dietary intake is expensive and time-consuming for both researchers and participants (17), this study aimed to collect the most detailed dietary intake data possible while being culturally appropriate and limiting the burden of participation for survey participants (16).

To assess dietary intake, adapted 24-h recalls were administered at both the household and individual levels, capturing foods consumed within the home (household level) and outside the home (individual level). This method was selected because, culturally, meals prepared within the household are typically prepared by the one main food preparer, who is thus best placed to report on the contents of these meals, and because meals consumed within the household are often shared among family members (16). Asking the household food preparer about consumption within the household also reduced the overall burden of survey participation for households. Previous research in Madagascar has found that shared household consumption is allocated proportionally to body weight (18).

For the individual-level survey, household members 16 years or older were asked directly about the foods they consumed outside the home. Individuals under the age of 16 were assumed to consume most of their food at or from home and thus were not asked about food consumption outside the home.

Both the household and individual surveys used a predetermined food list that included 240 culturally and nutritionally relevant foods, including almost a hundred marine fish and invertebrate families. The food list was developed based on foods reported as consumed in previous 24-h recalls conducted in similar populations in Madagascar, commonly consumed marine foods identified by local experts, and foods reported as consumed during focus groups with community members (16). Dietary intake was captured from midnight to midnight for the day immediately preceding the interview. If a food was identified as having been consumed, follow-up questions were asked to determine the portion size consumed, based on a predetermined list of portion sizes, and the number of portions consumed. For more details on the construction of the food list, please refer to Golden et al. (16). For a complete list of included foods, see Supplementary Table 1.

#### Nutrient composition database construction

A nutrient composition database was created for the calories in each food. Through cross-multiplying each food by its serving size and calorie content, we identified the number of calories per serving for each of the 240 foods included in the adapted 24-h recall. Sources of the nutrient composition database included past research from Madagascar (19), the Aquatic Food Composition Database (11), and other nutrient composition databases from Tanzania (20), the United States (21), and Australia (22).

#### Categorizing dietary data into food groups

The 240 foods consumed were categorized into culturally and nutritionally relevant food groups, adapted from the Global Diet Quality Score (23). The Global Diet Quality Score food groups were selected because they were developed using scientific evidence on the relationship between food intake and health outcomes related to undernutrition and overweight and obesity related health outcomes, and were designed for global use, including in low- and middle-income contexts (23). Foods were allocated to food groups based on the Global Diet Quality Score standards with four exceptions, (1) coconut was added to the ‘other fruit’ category because it was excluded from the Global Diet Quality Score food groups, (2) given the role of rice in the diet in Madagascar, rice was removed from the ‘refined grains and baked goods’ (now called non-rice refined grains) food group and moved into its own group, (3) due to relatively low intake, poultry and red meat were combined into a domesticated meat grouping, and (4) due to relatively low intake, the deep orange vegetables (only carrots in this analysis) and deep orange tubers (only sweet potatoes in this analysis) food groups were combined. Food groups consumed by less than 5% of the population (12 food groups) were excluded from the analysis, except for the category of poultry and red meat, which was included despite being consumed by less than 5% of the population because of its importance in the Westernization of diets. The 13 food groups included in this analysis are dark green vegetables, deep orange fruits, deep orange vegetables and tubers, liquid oils, sweets, white roots and tubers, non-rice refined grains, legumes, other fruits, corn, poultry and red meat, fish and marine invertebrates, and rice. See Supplementary Table 1 for a list of the foods included in each food group and the food groups that were excluded from the study.

#### Estimating individual dietary intake

Consumption from the two recalls (household and individual) was combined and allocated to the individual level, allowing the dietary pattern analysis to be conducted at the individual level. By conducting the analysis at the individual level, the analysis does not assume that all members 16 years and older of the same household are part of the same dietary pattern.

The following steps were used to identify the proportion of household-level intake to allocate to each individual. First, we identified the expected energy requirement of each individual (see below for details), and then the proportion of the household’s total expected energy requirement that the individual’s expected energy requirement represents. This proportion of the reported household dietary intake of each food group was then allocated to the individual. Next, the percentage of the diet made up of each food group for each individual was calculated. The steps were completed as follows:

1 *Calculate expected energy requirement:* An expected energy requirement was calculated for each individual based on their sex, age, and weight, using the *Recommended Dietary Allowances* (24) as the basis for expected energy intake estimates. The expected energy intake requirements assume a moderate level of physical activity for all individuals.
2 *Calculate remaining energy needs after accounting for intake outside the home:* The remaining energy expected to be obtained from within the household after accounting for intake outside the home was calculated by subtracting energy intake reported from outside the home from the expected individual energy requirement calculated in step one.
3 *Adjust for pregnancy and breastfeeding status:*

- The expected individual energy requirement was adjusted to account for breastfeeding by adding 500 calories to the expected individual energy requirement for individuals who reported breastfeeding (*Recommended Dietary Allowances* (24)).
- The expected individual energy requirement was adjusted for pregnancy status by adding 300 calories to the expected individual energy requirement for individuals reported to be in the second, third, or unknown trimester of pregnancy (n = 2 with an unknown pregnancy trimester) (*Recommended Dietary Allowances* (24)).

4 *Account for the presence of guests:* The presence of guests in the household on the survey day was accounted for by adding one-third of each guest’s expected individual energy requirement to the total expected household intake. This assumes that guests received one-third of their daily caloric needs from the household, or in other words, that they were present for one meal. As the exact age and weight of each guest were unknown, an estimated individual energy requirement was calculated using weighted averages of the expected energy requirements for their known age group and sex (Recommended Dietary Allowances (24)). See Supplementary Table 2 for estimated individual energy requirements used for guests.
5 *Identify the proportion of household intake to allocate to each individual:* The proportion of household intake to allocate to each member of that household was calculated by summing the adjusted expected individual energy requirement for each member of the household and guest (calculated in steps 1–5) to obtain the household total, and then dividing each individual’s remaining expected individual energy requirement by the household total. This proportion was then used to allocate household consumption of each food group to each individual for each wave of data collection.
6 *Add outside the home intake to allocated household intake:* Intake reported by each individual outside the home was then added to the estimated within-home consumption for each individual for each wave of data collection.
7 *Average intake over the 2 years:* To obtain a measure of typical dietary intake while limiting the impact of individual day-to-day variation in dietary intake (17), the average caloric intake for each food group for each individual was calculated across the number of dietary recalls they participated in (ranging from two to eight recalls, with an average of 7.1). For example, if the individual participated in six of the eight dietary recalls, their calorie intake for each food group and total caloric intake was averaged across the six dietary recalls.
8 *Calculate proportion of diet made up of each food group:* To limit the impact of differences in total caloric intake on the dietary pattern analysis (17), the estimated average calorie intakes for each food group for each individual resulting from step seven were made isocaloric by dividing intake for each food group by total caloric intake for each individual. For example, the average intake for the food group of fish and marine invertebrates was divided by the total average caloric intake to obtain an estimate of the percentage of the diet of that individual that is composed of fish and marine invertebrates.

After complete dietary intake profiles were created for each individual, we allocated individuals to category groups for each food group (tertile of positive consumption (i.e., zero consumption, or low, medium, or high tertiles of positive consumption)) based on the percentage of their diet contributed by each food group (25, 26). The use of categorical data was chosen to minimize the undue influence of outlier values in the reported dietary intake data (17).

#### Socio-demographic survey data

The socio-demographic survey consisted of two modules: one administered to the head of household and another to all household members. For those under 13, a caregiver completed the study on their behalf. The head of household survey covered sex, age, occupation, educational attainment, household income, and food security (as measured by the World Food Programs Coping Strategies Index) (27). Questions on individual sex, age, occupation, and income were included in the individual-level module. For more details on the socio-demographic survey, please refer to Golden et al. (16).

### Statistical analysis

As this analysis aims to understand, for the first time, dietary patterns in southwestern Madagascar, we employed a data-driven method to identify the patterns. Dietary patterns were identified using the Weighted Overfitted Latent Class Analysis (WOLCA) method (28). Latent Class Analysis (LCA) was selected because it is a finite mixture model clustering method that summarizes data from correlated categorical variables (in this case, food groups) into a few latent clusters (in this case, dietary patterns) (29, 30). WOLCA performs LCA to identify dietary patterns while accounting for survey design. Estimation of dietary pattern membership, the prevalence of the dietary patterns in the population, and the food group consumption level probabilities for each dietary pattern proceeds via the Bayesian paradigm using Markov chain Monte Carlo (MCMC) sampling, and a sparsity-inducing prior is used for data-driven selection of the number of dietary patterns (See Appendix: WOLCA Model Description, for details). Survey design is accounted for using a Bayesian pseudo-likelihood with a post-processing variance adjustment.

Due to stratification, study participants had an unequal probability of being sampled, so the WOLCA method was used to upweight (using inverse probability of sampling weights) each observation’s likelihood contribution proportional to the number of individuals they represent in the target population (31). The data also had clustering at the village (12 villages) and household (372 households) levels, which was accounted for by the WOLCA method using a post-processing variance adjustment (28).

The WOLCA method is ‘Overfitted’ because it starts with an intentionally high value that represents the maximum possible number of latent classes, then drops empty and unnecessary classes during the Bayesian estimation procedure, instead of relying on post-analysis model comparisons like the Akaike Information Criterion or Bayesian Information Criterion (28). By default, the WOLCA method excludes dietary patterns that comprise less than 5% of the population (28, 31). The “baysc” (Bayesian Survey Clustering) R package version 0.1.0 (32) was used for the analysis. The package does not currently allow for multiple MCMC chains to be run simultaneously, so three chains were run with different seeds for comparison. Throughout, weakly informative priors were used for the model parameters (See Appendix: WOLCA Model Description, for details). The WOLCA model was run for 20,000 iterations with a burn-in of 5,000 iterations for each of the three chains. Thinning was conducted every three iterations, and parameter estimates were summarized with the posterior median and 95% posterior intervals. All statistical analyses were conducted in R version 2025.05.0 + 496.

Multiple diagnostic plots were evaluated to assess model convergence, including a dendrogram, trace plots, and autocorrelation function (ACF) plot. The dendrogram was used to assess dietary pattern separation, with vertical distance (represented by vertical bars) between clusters (horizontal bars) indicating greater differentiation between dietary patterns. Trace plots were used to assess the stability of parameter estimation by displaying the estimated values at each iteration of the MCMC sampler. It indicated whether the chain successfully explored the possible parameter values and whether it then converged around a central estimate. The AFC plot was used to assess whether the MCMC iterations exhibited lingering autocorrelation or if autocorrelation quickly diminished toward zero, which would indicate efficient parameter estimation.

## Results

### Population characteristics

The overall population was estimated to be 52.6% (SE 1.3%) female, with 13.3% (SE 1.0%) aged zero to five, 37.1% (SE 1.6%) aged six to 18, and 49.6% (SE 1.5%) over the age of 18, resulting in an overall mean age of 23.3 (Table 1). The mean household size was 5.3 (SE 0.2). Overall, 36.4% (SE 4.3%) of household heads had no formal education, while 34.4% (SE 4.1%) had some primary education, and 29.2% (SE 4.9%) had some secondary education. Looking at occupations, 76.1% (SE 4.8%) of households participated in fishing, 34.6% (SE 3.5%) in aquaculture, and 28.7% (SE 3.6%) in crop-based agriculture.

**Table 1:** Estimated population size and socio-economic characteristics by dietary pattern.

Variable	Level	Traditional	Industrialized–transitioning	Traditional–undernourishing	Industrialized–undernourishing	Overall
Prevalence						
*N*: % (posterior SD %)		35.9 (11.1)	29.9 (13.5)	16.3 (5.2)	17.8 (11.2)	
*n*: %		37.8	26.3	18.6	17.3	
Individual characteristics						
Sex: % (SE)	Male	51.1 (2.2)	41.6 (2.6)	49.4 (3.4)	47.2 (2.6)	47.4 (1.3)
	Female	48.9 (2.2)	58.4 (2.6)	50.6 (3.4)	52.8 (2.6)	52.6 (1.3)
Age category: % (SE)	Child (0–5)	14.0 (1.7)	11.1 (2.0)	17.2 (4.4)	11.3 (2.4)	13.3 (1.0)
	Adolescent (6–18)	47.9 (2.5)	28.9 (4.1)	30.0 (4.2)	35.2 (4.0)	37.1 (1.6)
	Adult (19+)	38.0 (2.4)	60.0 (3.6)	52.8 (4.1)	53.4 (2.8)	49.6 (1.5)
Age: mean	Mean	20.3 (0.8)	27.5 (1.5)	21.8 (1.5)	24.0 (1.0)	23.3 (0.6)
Calorie intake tertile: % (SE)	Low	22.6 (2.8)	32.2 (4.3)	70.9 (4.8)	46.3 (7.8)	37.8 (2.7)
	Medium	32.7 (2.5)	32.7 (3.9)	20.1 (4.5)	30.8 (5.2)	30.2 (2.0)
	High	44.7 (3.1)	35.1 (3.4)	9.0 (2.2)	22.9 (4.6)	32.0 (2.1)
Household characteristics						
Household size (SE)	Mean	5.6 (0.2)	4.6 (0.3)	5.8 (0.4)	5.3 (0.9)	5.3 (0.2)
Household income tertile: % (SE)	Low	33.6 (4.6)	36.6 (7.2)	37.4 (6.8)	44.9 (9.6)	37.0 (4.3)
	Medium	37.0 (4.4)	30.2 (6.0)	31.6 (5.2)	27.6 (6.0)	32.5 (3.4)
	High	29.5 (4.2)	33.2 (5.5)	31.0 (6.0)	27.6 (6.2)	30.5 (3.2)
Per household member income tertile: % (SE)	Low	35.2 (4.6)	31.0 (7.4)	41.4 (6.7)	40.0 (10.1)	35.9 (4.2)
	Medium	36.7 (4.7)	31.6 (6.1)	36.3 (6.2)	25.0 (5.8)	33.2 (3.6)
	High	28.1 (3.7)	37.5 (5.8)	22.3 (4.3)	35.0 (7.1)	31.0 (3.1)
Head of household education level: % (SE)	None	41.1 (5.5)	24.5 (6.7)	41.9 (7.9)	43.5 (6.8)	36.4 (4.3)
	Some primary	33.9 (5.2)	33.1 (5.8)	32.2 (6.7)	42.1 (6.7)	34.4 (4.1)
	Some secondary	25.0 (5.3)	42.4 (7.4)	25.9 (8.9)	14.4 (4.4)	29.2 (4.9)
Coping strategies index tertile: % (SE)	Low	28.7 (4.9)	63.9 (5.8)	25.4 (7.0)	16.0 (4.2)	36.3 (4.4)
	Medium	35.8 (4.7)	25.8 (5.1)	25.3 (5.6)	38.7 (8.5)	31.5 (3.6)
	High	35.5 (4.8)	10.3 (2.5)	49.3 (6.9)	45.3 (9.2)	32.2 (3.7)
Fishing participation: % (SE)	No	14.0 (4.6)	41.6 (7.5)	21.1 (7.1)	17.5 (11.2)	23.9 (4.8)
	Yes	86.0 (4.6)	58.4 (7.5)	78.9 (7.1)	82.5 (11.2)	76.1 (4.8)
Aquaculture participation: % (SE)	No	63.6 (4.6)	69.0 (5.1)	71.1 (5.2)	57.2 (8.1)	65.4 (3.5)
	Yes	36.4 (4.6)	31.0 (5.1)	28.9 (5.2)	42.8 (8.1)	34.6 (3.5)
Crop-based agriculture participation: % (SE)	No	65.3 (4.8)	79.3 (5.9)	68.0 (6.3)	74.1 (6.2)	71.3 (3.6)
	Yes	34.7 (4.8)	20.7 (5.9)	32.0 (6.3)	25.9 (6.2)	28.7 (3.6)

Percentages in the second row (sample size, n) are unweighted. All other rows present survey-weighted estimates based on sampling weights, representing the estimated percentages (and standard errors, SEs) of the HIARA cohort population within each category. As a result, tertile groups may not correspond to exactly one-third of the population. For the first row (population size, N), SEs were derived from the Bayesian posterior samples of the class membership probabilities and represent the uncertainty in the estimated class proportions. For other variables, frequentist SEs were calculated assuming fixed latent class assignments and therefore likely underestimate the true uncertainty that would arise if latent class misclassification were accounted for.

### Validating dietary patterns

Exploration of the diagnostic plots indicates the clustering process was successful. Specifically, investigation of the dendrogram shows reasonable separation (vertical distance) between the final four clusters, suggesting that they represent distinct dietary patterns in the population (See Supplementary Figure 1). Further investigation of the trace plots reveals reasonable convergence around the final prevalence estimates for each dietary pattern, indicating that the sampler successfully explored the possible parameter values (See Supplementary Figure 2). Finally, investigation of the ACF plots did not show persistent autocorrelation as the autocorrelation estimates quickly decayed toward zero (see Supplementary Figure 3). The dietary patterns identified using different seeds were similar.

### Identified dietary patterns

**Figure 1:**
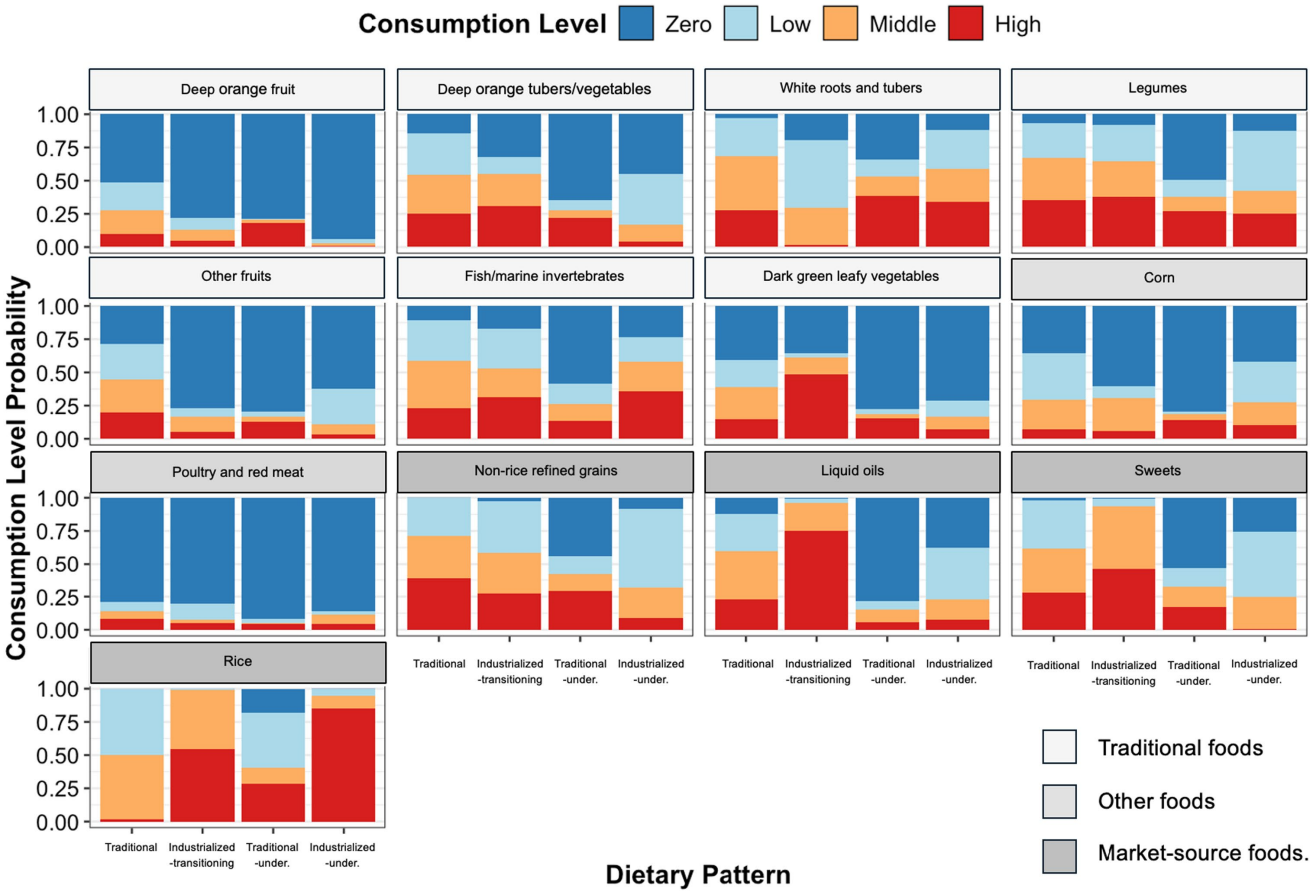
Consumption level breakdown by dietary pattern and food groups. On the X-axis, ‘Traditional’ represents the traditional dietary pattern, ‘Industrialized-transitioning’ represents the industrialized-transitioning dietary pattern, ‘Traditional-under.’ represents the traditional-undernourishing dietary pattern, and ‘Industrialized-under.’ represents the industrialized-undernourishing dietary pattern. The Y-axis shows the estimated consumption level probability for each dietary pattern. Color coding represents the different consumption levels (zero consumption, low tertile of the percentage of diet, medium tertile of the percentage of diet, or high tertile of the percentage of diet) of individuals estimated to be part of the corresponding dietary pattern. The y-axis represents the probability of each consumption level.

The dietary pattern analysis identified four distinct dietary patterns among the individuals represented by the HIARA cohort. These four dietary patterns aligned well with three (traditional, mixed, and undernourishing) of the four previously hypothesized dietary patterns (traditional, mixed, overnourishing, and undernourishing) theorized to result from social-ecological traps in coral-reef-based food systems (11) (Figure 1). Figure 1 provides a detailed breakdown of the different consumption levels (zero, or low, medium, or high percent of the diet tertile) among those in each dietary pattern for each food group. The 95% posterior intervals can be seen in Supplementary Table 3. Food groups shown first in Figure 1, namely deep orange fruits, deep orange tubers and vegetables, white roots and tubers, legumes, other fruits, fish and marine invertebrates, and dark green leafy vegetables are considered to be more traditional foods food groups for southwestern Madagascar, while those shown last, namely non-rice refined grains, liquid oils, sweets, and rice, are considered to be less traditional and more typically sourced from the market.

We characterized the first dietary pattern as a traditional diet in coastal southwestern Madagascar, given its higher dietary diversity and higher contributions of traditional foods, including deep orange fruits and tubers, white roots and tubers, legumes, fish and marine invertebrates, and other fruits (Figure 1). The diet cannot be considered entirely traditional due to the modest intake of non-rice refined grains, rice, and sugar; however, it is more traditional than the other three identified dietary patterns. We characterized the second dietary pattern as an industrialized-transitioning diet because it contains a high intake of traditional foods such as fish and marine invertebrates and legumes, but is generally less diverse and more associated with foods procured from the market, including liquid oils, sweets, and rice. This is akin to the mixed dietary pattern previously hypothesized (11). We characterized the third dietary pattern as a traditional-undernourishing diet, given that it had the lowest average calorie intake (Table 2) and consisted primarily of nutrient-poor white roots and tubers, non-rice refined grains, and rice. Lastly, we characterized the fourth dietary pattern as an industrialized-undernourishing diet, as it relied heavily on nutrient-poor rice (63% of calories, Table 2) and consisted of more market-source foods, including rice, liquid oils, and sugar, than the other traditional-undernourishing dietary pattern. None of the identified dietary patterns were characterized as fully industrialized or westernized, as none of the dietary patterns were fully dominated by highly processed or sugar sweetened foods beyond the high consumption of white rice.

**Table 2:** Percent of the diet composed of each food group [Mean (SE)].

Food group	Traditional	Industrialized- transitioning	Traditional- undernourishing	Industrialized- undernourishing
Traditional food groups				
Deep orange fruits	0.4 (0.2)	0.1 (0.1)	2.3 (1.3)	0.0 (0.0)
Deep orange tubers and vegetables	0.1 (0.0)	0.4 (0.0)	1.6 (1.2)	0.0 (0.0)
White roots and tubers	**18.7 (1.6)**	**14.5 (2.5)**	**21.6 (3.5)**	**13.3 (2.7)**
Legumes	**6.2 (0.7)**	4.4 (0.6)	4.0 (1.9)	2.8 (0.6)
Other fruits	**7.0 (1.7)**	1.3 (0.4)	4.1 (1.5)	1.2 (0.4)
Fish & marine invertebrates	**5.9 (0.5)**	5.5 (0.6)	3.1 (0.8)	**6.5 (0.9)**
Dark green leafy vegetables	0.8 (0.2)	2.8 (0.9)	2.0 (0.6)	0.8 (0.2)
Other food groups				
Corn	2.6 (0.5)	0.8 (0.3)	1.2 (0.8)	2.3 (1.2)
Poultry and red meat	0.3 (0.2)	0.2 (0.1)	0.5 (0.4)	0.5 (0.2)
Market-source food groups				
Non-rice refined grains	**16.4 (0.0)**	**18.0 (1.5)**	**16.2 (3.2)**	**6.0 (1.1)**
Liquid oils	2.9 (0.3)	**8.6 (0.9)**	1.7 (0.8)	1.9 (0.5)
Sweets	4.3 (0.4)	3.7 (0.2)	2.9 (0.8)	0.8 (0.2)
Rice	**33.0 (1.5)**	**39.2 (1.9)**	**37.8 (3.5)**	**63.4 (3.6)**
From food groups not included	0.0	0.0	0.0	0.0
Estimated calorie intake	**1831.8 (38.6)**	1615.5 (52.9)	947.3 (52.2)	1452.3 (63.6)

Numbers in bold represent more than 5 % of the diet. Estimates for the average percent of the diet for each food group were weighted to represent the population in the regions using inverse probability of sampling weights.

### Individual characteristics of dietary patterns

Membership in the dietary patterns also varied by sex, with the overall population 52.6% female (SE 1.3%), but with those in the traditional dietary pattern only 48.9% female (SE 2.2%), those in the industrialized-transitioning dietary pattern 58.4% female (SE 2.6%), and those in the two undernourishing dietary patterns 50.6–52.8% female (Table 1). Those in the industrialized-transitioning dietary pattern were the oldest on average (mean, 27.5 years; SE 1.5), while those in the traditional dietary pattern were the youngest (mean, 20.3 years; SE 0.8). The age breakdown also differed by dietary pattern, with a low of 38.0% (SE 2.4%) of those in the traditional dietary pattern over the age of 18 and a high of 60.0% (SE 3.6%) of those in the industrialized-transitioning dietary pattern over the age of 18. A low of 28.9% (SE 4.1%) of those in the industrialized-transitioning dietary pattern were between the ages of six and 18, while a high of 47.7% (SE 2.5%) of those in the traditional dietary pattern were between the ages of six and 18. A low of 11.1% (SE 2.0%) of those in the industrialized-transitioning dietary pattern were between the ages of zero and five, while a high of 17% (SE 4.4%) of those in the traditional-undernourishing dietary pattern were between the ages of zero and five.

Those in the traditional (44.7%, SE 3.1%) and industrialized-transitioning (35.1%, SE 3.4%) dietary patterns were more likely to be in the highest tertile of calorie intake, while those in the traditional-undernourishing (9.0%, SE 4.6%) and industrialized-undernourishing (22.9%, SE 4.6%) dietary patterns. Those in the traditional-undernourishing dietary pattern were most likely to be in the lowest tertile of calorie intake (70.9%, SE 4.8%).

### Household characteristics of dietary patterns

The traditional dietary pattern was estimated to include 35.9% (SD 11.1%) of individuals, the industrialized-transitioning pattern 29.2% (SD 13.5%), the traditional-undernourishing dietary pattern 16.3% (SD 5.2%), and the industrialized-undernourishing dietary pattern 17.8% (SD 11.2%) (Table 1; and see Supplementary Figure 4 for histogram of dietary pattern membership estimates).

Fishing predominated, with 76.1% (SE 4.8%) of the population being from households that participated in fishing. Among them, a disproportionately higher number of households (86.0%, SE 4.6%) in the traditional dietary pattern fished, and a disproportionately lower number of households (58.4%, SE 7.5%) in the industrialized-transitioning dietary pattern fished. Approximately 80% of those in the two undernourishing dietary patterns engaged in fishing, showing greater similarity to those in the traditional dietary pattern over the industrialized-transitioning dietary pattern with respect to fishing participation. In contrast, household participation in crop-based agriculture was more evenly distributed across the dietary patterns, with 28.7% (SE 3.6%) of the overall population, 34.7% (SE 4.8%) of the traditional dietary pattern, 20.7% (SE 5.9%) of the industrialized-transitioning dietary pattern, 32.0% (SE 6.3%) of the traditional-undernourishing dietary pattern, and 25.9% (SE 6.2%) of industrialized-undernourishing dietary pattern, coming from households that participated in crop-based agriculture.

Examining the relationship between household income and dietary patterns, we observed that the industrialized-transitioning dietary pattern had the highest per household member income, with 33.2% (SE 5.5%) in the highest tertile of per household member income. Those in the traditional-undernourishing dietary pattern had the lowest per household member income, with 41.4% (SE 6.7%) falling into the lowest tertile of per household member income and only 22.3% (SE 4.3%) in the highest tertile of per member household income.

Overall, most heads of household had no formal education (36.4%, SE 4.3%) or some primary education (34.4%, SE 4.1%). However, a defining feature of the industrialized-transitioning dietary pattern was that it had the largest proportion of heads of household who had completed some secondary education (42.4%, SE 7.4%) and the lowest proportion of heads of household who had completed no formal education (24.5%, SE 6.7%). More than 40% of households from the other three dietary patterns had heads of households who had completed no formal education (41.1–43.5%).

The Coping Strategy Index (CSI) was used as a measure of household food insecurity, with higher scores indicating a lower level of household food security (27). Individuals in the industrialized-transitioning dietary pattern (63.9%, SE 5.8% low CSI score tertile and 10.3%, SE 2.5% high CSE score tertile) were relatively more food secure than those in the traditional-undernourishing (49.3% (SE 6.9%) high CSI score tertile) and industrialized-undernourishing (45.3% (SE 9.2%) high CSI score tertile) dietary patterns. Those in the traditional dietary pattern fell in the middle with 35.5% (SE 4.8%) in the high CSI score tertile and 28.7% (SE 4.9%) in the low CSI score tertile.

### Differences in intake by dietary pattern

Across the dietary patterns, differences were observed in the percentage of the diet comprised of each food (Table 2). Those in the traditional dietary pattern consumed most of their diet from white roots and tubers, non-rice refined grains, legumes, other fruits, fish and marine invertebrates, and rice. Those in the industrialized-transitioning dietary pattern consumed most of their diet from liquid oils, white roots and tubers, non-rice refined grains, and rice. Those in the traditional-undernourishing dietary pattern consumed most of their diet from white roots and tubers, non-rice refined grains, and rice. Lastly, those in the industrialized-undernourishing dietary pattern consumed most of their diet from white roots and tubers, non-rice refined grains, and rice. While those in all four dietary patterns consumed staples including white roots and tubers, non-rice refined grains, corn, and rice in high amounts, the proportion of the diet made up of staples varied by dietary pattern. Staples made up 71% of the diet for those in the traditional dietary pattern, 72% of the diet for those in the industrialized-transitioning dietary pattern, 77% of the diet for those in the traditional-undernourishing dietary pattern, and 84% of the diet for those in the industrialized-undernourishing dietary pattern.

Differences can also be observed in the total number of calories consumed overall and by food group (Table 2). Calorie intake was estimated to be 1831.8 (SE 38.6) calories for those in the traditional dietary pattern, 1615.5 (SE 52.0) calories for those in the industrialized-transitioning dietary pattern, 947.3 (SE 52.2) calories for those in the traditional-undernourishing dietary pattern, and 1452.3 (SE 63.6) calories for those in the industrialized-undernourishing dietary pattern.

## Discussion

### Identified dietary patterns

The results of this dietary pattern analysis provide important insights into the dietary patterns present in southwestern Madagascar and describe the socio-economic characteristics common to each dietary pattern. These results inform our understanding of how social and ecological forces may influence food systems and dietary futures in southwestern Madagascar through dietary patterns. Previous work predicted that social-ecological traps would result in four distinct dietary patterns in coral-reef-based food systems: a traditional dietary pattern characterized by high intake of traditional foods, a mixed pattern characterized by intake of both traditional and market-source foods, an overnourishing dietary pattern characterized by high intake of market-source foods, and an undernourishing dietary pattern characterized by low intake of nutrient-dense traditional or market-source foods (11). In southwestern Madagascar, we identified three of the four hypothesized dietary patterns: a traditional dietary pattern, an industrialized-transitioning (mixed) dietary pattern, and two undernourishing dietary patterns, one characterized by a high reliance on staples and the other by a high dependence on staples, as well as higher consumption of other market-source foods compared to the other undernourishing dietary pattern.

A fully industrialized or overnourishing dietary pattern was not identified, as none of the identified dietary patterns were fully dominated by high intake of highly processed or sugar-sweetened foods, beyond the high consumption of white rice. However, the industrialized-transitioning dietary pattern is considered a shift toward an overnourishing dietary pattern and away from a traditional diet. This aligns with findings from Madagascar as a whole that show that while the prevalence of overweight and obesity are on the rise, especially among adults (4), the prevalence of these health outcomes remains low in comparison to the global north (33), again suggesting that Madagascar is earlier in the nutrition transition (Popkin (20)). This early stage of the nutrition transition may be due to the high levels of poverty in Madagascar (34), as poverty can constrain financial access to market-source foods, even when they are physically available”.

### Quantity and quality of intake by dietary pattern

Of the four dietary patterns identified in the region, two—traditional and industrialized-transitioning—were characterized as higher-quality diets due to their higher dietary diversity, while the two undernourishing dietary patterns were considered poor-quality diets for the region due to their lower dietary diversity, high reliance on nutrient-poor staples, and low calorie intake.

The two undernourishing dietary patterns aligned with the undernourishing dietary pattern previously hypothesized (11), described as having inadequate quality and potentially inadequate quantity of intake. Both the undernourishing dietary patterns identified here rely heavily on the intake of nutrient-poor white roots and tubers, non-rice refined grains, corn, and rice, with staples contributing the vast majority to overall caloric consumption (77–84%) and thus can be considered to represent poor quality intake. Examining the quantity of dietary intake, individuals in the traditional-undernourishing dietary pattern had the lowest calorie intake of the three dietary patterns [947.3 (SE 52.2)], and those in the industrialized-undernourishing dietary pattern had the second lowest intake [1452.3 (SE 63.6)]. Even if this estimate of calorie intake for the traditional-undernourishing dietary pattern is underestimated by the typical 10 to 20% (17, 35), it likely represents inadequate calorie intake for meeting the population’s basic energy needs. The two undernourishing dietary patterns thus align with the ‘potentially’ adequate quantity of dietary intake previously hypothesized for the undernourishing dietary pattern in a corral-reef-based food system (11). Differences in consumption of rice and other foods between the two undernourishing dietary pattern groups may result from differences in physical or financial access to these foods, or from different prioritization of certain foods or food groups. For instance, white rice may be a desirable food for some individuals due to dietary preferences or because it is seen as socially or aspirationally desirable. The hypothesized undernourishing dietary pattern was described as resulting from a loss of access to traditional foods, combined with limited or no access to sufficiently nutritious foods from the market. This social-ecological context thus traps individuals into an undernourishing dietary pattern - such as the two identified here as present in southwestern Madagascar.

While the previously hypothesized traditional dietary pattern was expected to have both adequate quantity and quality of intake, the mixed dietary pattern, akin to the industrialized-transitioning dietary pattern identified here, was predicted to have adequate quantity but potentially inadequate quality of intake (11). The traditional dietary pattern identified here was the most diverse, with the largest number of food groups comprising 5% or more of the diet (Table 2). While dietary diversity is not a perfect proxy for diet quality (36), the diversity, coupled with the nutrient density of the food groups consumed, including legumes, fruits, and fish and marine invertebrates, suggests the traditional dietary pattern is the highest quality pattern of the four identified patterns. Those in the traditional dietary pattern also relied more heavily on traditional staples, including white roots and tubers, and consumed the lowest percent of their diet from rice. While the industrialized-transitioning dietary pattern appears to represent a lower-quality diet compared to the traditional diet due to its lower dietary diversity and higher reliance on nutrient-poor food groups, including non-rice refined grains and rice, it was still more diverse than the two undernourishing dietary patterns.

Looking at the quantity of dietary intake, we see that the traditional dietary pattern had the highest calorie intake of the four patterns [1831.8 (SE 38.6)], that the industrialized-transitioning [1615.5 (SE 52.0)] and industrialized-undernourishing 1452.3 (SE 63.6) dietary patterns had medium calorie intakes, and that the traditional-undernourishing dietary pattern had the lowest calorie intake [947.3 (SE 52.2)]. Understanding how these calorie intakes translate to an adequate quantity of intake necessitates further study of the association between the dietary pattern membership and nutrition-related health outcomes, specifically underweight status. Differences in physical activity may also have contributed to the difference in calorie intake in this population, as physical activity is considered the most significant driver of between-person differences in energy intake (20). The role of occupational or recreational physical activity as a driver of calorie intake means that individuals in the traditional dietary pattern may have consumed more calories due to higher physical activity levels, especially in this population, where many participants work in physically demanding occupations, such as fishing and crop-based agriculture.

Despite these differences in quality and quantity of dietary intake, all four identified dietary patterns had similarities, including a low intake of whole grains, fruits and vegetables, and nuts and seeds, food groups that have been identified as important for human health (37, 38). Low intake of these nutrient-dense food groups may be due to limited financial or physical access to these foods, as nutrient-dense foods can be both unaffordable (39) and unavailable (40) in Madagascar. The low intake of whole grains, fruits and vegetables, and nuts and seeds across all four dietary patterns means that, while the traditional dietary pattern represents a diet that is comparatively higher in quality and quantity, it is likely inadequate for ideal health.

### Social and environmental context and drivers of dietary patterns

The dietary patterns we have described are shaped by their social and environmental context. For example, individuals in the traditional and industrialized-transitioning dietary patterns were least likely to be in the low tertile of household income, whereas poverty limited food access for those in the traditional-undernourishing and industrialized-undernourishing dietary patterns, as 40–41% were in the lowest tertile of per member household income and a limited income can restrict the financial accessibility of food (39, 41). The undernourishing dietary patterns were both characterized by a high intake of relatively more affordable staples, such as white roots and tubers, non-rice refined grains, and rice, rather than animal-sourced foods like seafood, poultry and red meat, or other protein-rich foods like legumes (39, 40).

The sex and age composition of the dietary patterns also varied, with those in the industrialized-transitioning dietary pattern the most likely to be female [58.4% (SE 2.6%)], while those in the traditional dietary pattern were the least likely to be female [48.9% (SE 2.2%)]. These differences suggest women in Madagascar may be at greater risk for non-communicable diseases, as intake of ultra-processed market source foods is associated with an increased risk of such diseases, including obesity, type 2 diabetes, and cardiovascular disease (42). The diets of women deserve special consideration, as they not only have a direct impact on the health of women but also the health of their future or current children, if they are or become pregnant or start breastfeeding (43).

The highest percentage of adults was in the industrialized-transitioning dietary pattern, and the lowest percentage was observed in the traditional dietary pattern, suggesting that adults were less likely to consume a traditional diet and more likely to consume a industrialized-transitioning dietary pattern, which can be seen as a step toward an overnourishing, fully industrialized or Westernized dietary pattern (11). The traditional dietary pattern had the highest percentage of adolescents aged 6 to 19, whereas the traditional-undernourishing dietary pattern had the highest percentage of children aged 0 to 5. This difference suggests a cultural or access difference in the foods provided or available to those aged 6 to 18 and those aged 0 to 5. Further research is needed to understand the cultural drivers of diets in this context, namely how households make choices about food allocation, consumption, production, and purchasing under economic and/or environmental constraints.

Beyond the socio-economic factors that drive dietary patterns, these patterns are also shaped by their environmental context. Particular occupations will be more or less sensitive to ongoing and future environmental changes because their sectors rely more intimately on functioning ecological conditions. Specifically, household participation in fishing, aquaculture, and crop-based agriculture may be particularly sensitive to changing local ecological conditions, therefore pushing the dietary patterns of those who participate in such occupations toward nutritional vulnerability. Households that participate in fishing are affected by cyclones and coral bleaching, which have been occurring with increased frequency in Madagascar (8, 16, 44, 45), and overfishing and local ecological damage driven by local and international demand for marine source foods (16, 44, 46–48), and potentially will be impacted by the poleward shift of marine species and altered nutrient content of fish driven by rising sea temperatures (49). Households that participate in agriculture are particularly subject to recurrent drought conditions, which have already been identified as drivers of food insecurity in Madagascar (7). Given that those in the traditional-undernourishing dietary patterns were highly reliant on agricultural products for their dietary intake, with white roots and tubers, making up 13–22% of their intake and rice making up 38–63% of their intake, and were seemingly unable to access diverse traditional of market source foods, decreasing agricultural production may make it even harder for them to meet their basic calorie needs. Given that individuals in the traditional dietary pattern were the most likely to participate in fishing and crop-based agriculture, they may be pushed by these changing environmental forces into an overnourishing dietary pattern if they have financial access to market-source foods or an undernourishing dietary pattern if they do not.

While the findings of this study represent 2 years and one region, they can also be seen as a harbinger of what is to come if the forces driving dietary patterns in Madagascar are not first understood and then addressed. Given the current environmental and socio-economic trajectories, a shift to over- and undernourishing diets is likely to occur across Madagascar. Currently, an estimated 80% of the population is employed in predominantly smallholder agriculture (9, 50), and 18% of households are in occupations related to fishing and aquaculture (INSTAT (51)). These occupations are likely to be negatively impacted by climate change, resulting in lower availability and accessibility of traditional foods (7, 10, 11, 49). While those with income may attempt to replace their traditional food intake with market-sourced foods, market access is not always reliable in Madagascar due to limited infrastructure (52). Financial access to market-sourced foods will also be limited for many due to limited income, as alternative income-earning opportunities are limited in many regions (52, 53), and extreme poverty is common, with 81% of the population living below the international poverty line (50). As environmental change continues to limit access to traditional foods, market-source foods may not be a viable replacement for large segments of the population (13), who will instead be forced to transition from traditional and industrialized-transitioning dietary patterns to undernourishing ones. An increased prevalence of an under-nourishing dietary pattern could further exacerbate the prevalence of undernutrition-related health outcomes in Madagascar, which is already considered one of the most chronically undernourished countries (4). For those who can replace traditional foods with market-source foods, the industrialized-transitioning dietary pattern can be seen as a step toward adopting a fully industrialized or Westernized overnourishing dietary pattern that results from an over-reliance on highly processed, energy-dense, market-source foods, further driving the nutrition transition in Madagascar and increasing nutrition-related non-communicable disease health outcomes (11, 54). This nutrition transition, however, is not seen as inevitable if political and civil society action is taken (55).

### Limitations

While this study has many strengths, including the use of longitudinal dietary intake data from an understudied population and the use of innovative methods of dietary pattern analysis that account for stratification and clustering of data, it is also subject to limitations. Dietary pattern analysis is typically open to bias due to the use of *post hoc* analysis to determine the number of dietary patterns; the WOLCA method used in this study is not subject to this limitation. Dietary intake data from 24-h recalls are also subject to several forms of bias, including potential misreporting of consumption by participants, day-to-day variation in intake, as well as differences in serving sizes or the nutritional composition of foods reported as consumed compared to those in the nutrient composition database (20). To limit the impact of day-to-day variation on this analysis, the dietary intake data provided for each individual were averaged across all of their surveys, and individuals were only included if they participated in more than one recall during follow-up. Despite this attempt to address the impact of day-to-day variation, bias may persist due to differences in the types of individuals who participated in varying numbers (2–8) of the surveys. Lastly, household dietary intake was allocated to individuals based on age, sex, weight, pregnancy, and breastfeeding status categories, a moderate activity level was assumed for everyone, guests were assumed to take in one-third of their estimated daily caloric needs from the household, and individuals under the age of 16 were assumed to consume most of their food from or within the home, all of which may not be accurate.

## Conclusion

Understanding the impacts of social and ecological forces in a coral-reef-based food system, such as those found in southwestern Madagascar, and how to address them, necessitates an understanding of the dietary patterns present and the individuals who follow them. Four dietary patterns were identified in southwest Madagascar, with the traditional-undernourishing dietary pattern including a high percentage of young children, and the industrialized-transitioning dietary pattern containing the highest percentage of adults and females. Those in the traditional dietary pattern were the most likely to fish and participate in crop-based agriculture, occupations that are both environmentally dependent. Understanding the dietary patterns of these groups helps us understand the nutritional risks they may face. Knowing who faces what risks is crucial, given the high levels of malnutrition already present in Madagascar, the country’s extreme susceptibility to climate change, and its high reliance on traditional foods and occupations that are likely to become even less reliable as the impacts of climate change intensify.

## Data Availability

The datasets presented in this article are not readily available because the dataset includes individualized health outcomes and characteristics. Requests to access the datasets should be directed to golden@hsph.harvard.edu.
