# Supplementary material for: A social-ecological trap theory-informed investigation of dietary patterns in southwestern Madagascar
Source: Front Nutr. 2026 Feb 17;13:1745233. doi: 10.3389/fnut.2026.1745233 (PMC12953102; doi:10.3389/fnut.2026.1745233)
Supplement: Supplementary file 1 [file Data_Sheet_1.docx]

*Appendix*

**Supplement One**: WOLCA Model Description

Weighted overfitted latent class analysis (WOLCA; Wu et al., 2024) was used to derive the dietary patterns in this manuscript. WOLCA is a Bayesian mixture model designed to uncover unobserved subgroups within correlated categorical data. It can be seen as providing two main extensions to traditional latent class analysis (LCA) by: 1) accounting for sample survey design so that results are representative at the population level (the "weighted" component); and 2) allowing for the number of patterns to be determined by the data using a sparsity-inducing prior within a Bayesian framework (the "overfitted" component).

We define some notation to describe our data setting:

- $n$ denotes the number of individuals in the sample, equal to 1,702 here.
- $J$denotes the number of food groups, equal to 13 here.
- $K$denotes the number of latent classes (i.e., dietary patterns) to be estimated from the data.
- $R_{j}\in\left\{ 0,1 \right\}$ denotes the number of food consumption levels in a given food group $j$, equal to 4 (“Zero”, “Low”, “Middle”, “High”) for all food groups here.
- $x_{ij}\in\{1,\ldots, R_{j}\}$ is an individual $i$’s observed consumption level for a food group $j$, for $i=1,\ldots,n$and $j=1,\ldots,J$.

The following parameters and latent variables are estimated from the WOLCA model:

- $c_{i}$ is the latent class (i.e., dietary pattern) assignment variable for individual $i$, $i=1,\ldots,n$. $c_{i}$ assigns each individual to exactly one of the $K$ dietary patterns.
- $\pi_{k}$is the class membership probability parameter for the latent class $k$ $k=1,\ldots,K$. This specifies the prevalence of the latent classes in the population.
- $\theta_{jkr}$is the item response probability parameter for food group $j$, latent class $k$, and consumption level $r$, $j=1,\ldots,J,$ $k=1,\ldots,K$, and $r=1,\ldots,R_{j}$. $\theta_{jkr}$ is specific to each latent class and provides information on the probability that an individual assigned to that class consumes a given food group $j$ at a given consumption level $r$. For each latent class, the consumption level with the highest probability is typically used to form the pattern profile. For instance, if food groups are consumed at four consumption levels: “Zero”, “Low”, “Middle”, and “High”, then a pattern profile is formed from the most probable consumption level for each food item among those belonging to that class, and these pattern profiles can be interpreted as dietary patterns.

As with LCA, WOLCA relies on the local independence assumption that items are independent conditional on dietary pattern assignment, and the global clustering assumption that individuals assigned to the same pattern share behaviors for all food items.

Equation (1) below shows the individual-specific complete-data likelihood for categorical dietary data under the WOLCA model:

$\prod_{k=1}^{K} \{\pi_{k}{\prod_{j=1}^{J} \prod_{r=1}^{R_{j}} \theta_{jkr}^{I\left( x_{ij}=r \right)}\}}^{I\left( c_{i}=k \right)}$. (1)

A key advantage of WOLCA is its ability to incorporate survey design features. In particular, when individuals are selected into the sample with unequal selection probabilities, survey sampling weights can be incorporated into parameter estimation to make results representative at the population level. This is done by up-weighting each individual's likelihood contribution in proportion to the number of individuals they represent in the population. This weighted pseudo-likelihood is then combined with priors for the parameters in order to obtain the posterior distribution of the parameters, weighted to be representative of the population. Equation (2) below shows how the weighted pseudo-likelihood is incorporated into the posterior update, letting $\Theta$ denote all parameters, $D$ denote the observed data, and $\tilde{w}_{i}$ denote the survey weight for individual $i$, normalized so that the sum of all survey weights is the sample size.

$p_{wtd}\left( \Theta| D \right)\propto p\left( \Theta\right)\prod_{i=1}^{n} p\left( D_{i} | \Theta\right)^{\tilde{w}_{i}}$ (2)

Estimation of the parameters proceeds under a Bayesian framework, where the posterior distribution of the parameters is estimated using a Markov chain Monte Carlo (MCMC) Gibbs sampling algorithm. For accurate variance estimates that account for survey design elements such as clustering and stratification, a post-processing variance adjustment is used to obtain adjusted posterior intervals that achieve approximately nominal coverage.

We use the following priors during parameter estimation:

$$\left( \pi_{1},\ldots,\pi_{K} \right)\sim Dirichlet\left( \frac{1}{K},\ldots,\frac{1}{K} \right)$$

$$\left( \theta_{jk1},\ldots,\theta_{jkR_{j}} \right)\sim Dirichlet\left( 1,\ldots,1 \right) \text{for }j=1,\ldots,J,k=1,\ldots,K.$$

The prior for the item response probabilities $\theta_{jkr}$ is a weakly informative prior that allows for flexibility in estimation. The prior for the class membership probabilities $\pi_{k}$ is a sparsity-inducing prior, adapted from the Bayesian nonparametrics literature, that enables the number of latent classes to be automatically selected by the data. This provides an advantage over traditional LCA, which the number of latent classes is typically selected post-hoc after multiple model fits and using some model selection criteria. For the sparsity-inducing prior, the number of latent classes is set to a conservatively high number (e.g., $K=30$), and then empty and unnecessary classes automatically drop out during the estimation process. The final number of classes, $K_{final}$, is determined by removing duplicate classes and retaining only those exceeding a prespecified threshold size, set to 0.05 for this study. This removes any classes that are observed in less than 5% of the population.

More details on the WOLCA method can be found in Wu et al. (2024) and Wu et al. (2025).

**Supplementary Table 1:** Food group composition

| **Food group** | **Food items** |
| --- | --- |
| 1. Dark green leafy vegetables | anamalahy, anamamy, moringa, watercress, anatsonga, petsay, squash leaves, cassave leaves, chayotte leaves, sweet potatoes leaves, taro leaves, tisam, sodesiny, epinard, letus, other dark green vegetable |
| 1. Deep orange fruits | malagasy melon, kakis, mangoes, green papaya, ripe papaya |
| 1. Deep orange tubers and deep orange vegetables | carrot, sweet potatoes |
| 1. Fish and marine invertebrates | cacharinus, centrophoridae, albulidae, gerreidae, monacanthidae, siganidae, xiphiidae, lutjanidae, acanthuridae, polynemidae, belonidae, hemiramphidae, fistulariidae, nemipteridae, aulostomidae, ephippidae, scaridae, apogonidae, diodontidae, tetraodontidae, caracanthidae, centrarchidae, centriscidae, monodactylidae, turtle, mobulidae, rajidae, chaetodontidae, lethrinida, pomacentridae, holocentridae, fiambondro, plotosidae, acanthuridae naso, mullidae, caesionidae, ophichthidae, clupeidae, pempheridae, echeneidae, terapontidae, pinguipedidae, kyphosidae, sparidae, dichistiidae, synodontidae, zanclidae, priacanthidae, pomacanthidae, scorpaenidae, muraenidae, bothidae, cynoglossidae, soleidae, carangidae, torpedinidae, labridae, leiognathidae, combridae, serranidae, sphyraenidae, cirrhitidae, microdesmidae, molidae, ophidiidae, peristediidae, psettodidae, rhinobatidae, blenniidae, gobiidae, ostraciidae, tetrarogidae, platycephalidae, callionymidae, congridae, mugilidae, cacharinus, haemulidae, exocoetidae, dactylopteridae, chirocentridae, balistidae, syngnathidae, ampozo, angeliky, babaky, regalecidae, tsotso, other fish, octopus, sepia, loligo, charonia, murex fasciolaria, anadara, scylla, tridacna, lambis, palunirus, tripneustes, pyrasus, pinctada, isognomon, atrina, pinna, aristeidae, holothuria, other marine invertebrates |
| 1. Legumes | green beans, peas, white beans, lima beans, tsiasisa, black eyes peas, bambara bean, mung pea, antaky, bobon kapiky, other bean/legume |
| 1. Liquid oils | oil, oil added to sauce |
| 1. Other fruits | kimoky, kononoky, fried banana, date palm, lemon, sugar cane, passion fruit, guava, green banana, ripe banana, tamarind, coconut, lamprey, litchis, pineapple, jujuba, peach, apple, raketa, sakoa, tsinefo, watermelon, vontaka, avocado, jevi, other fruit |
| 1. Non-rice refined grains | biscuit, cacapigeon, compose, lasopy, misao, paty instantané, spaghetti, macaroni, mokary, mofo dipaina, beignet greens, banana tempura, makasaoky, bokoboko, sambosa nem, other bread |
| 1. Rice | rice |
| 1. Sweets | sugar added to coffee, sugar added to tea |
| 1. White roots and tubers | potatoes, dry cassava, fresh cassava, taro, yam, wild tuber, oviala, piky, balo, other tuber, petisy, chips (potato) |
| 1. Corn | corn |
| 1. Poultry and red meat | akanga, chicken meat, organ liver, mulard, duck, goose, muscovy duck, turkey, meat, kabab, henan soy, pork, zebu, lamb, goat, other organ meat. Also included tortoise (consumed two times total) and cricket (included 1 time total). |
| **Not included due to low intake (<5% of population)** | |
| 1. Alcohol | wine, beer, alcohol, other alcohol |
| 1. Citrus fruits | mandarin, orange |
| 1. Cruciferous vegetables | cabbage, cauliflower, red cauliflower |
| 1. Eggs | eggs |
| 1. Low-fat dairy | NA |
| 1. High-fat dairy | cow milk, lamb milk, goat milk, yogurt, habobo, cheese, other milk, milk added to coffee, milk added to tea |
| 1. Other vegetables | endive, eggplant, cucumber, zucchini, mody, chayote, tomatoes, green onions, squash, other vegetable |
| 1. Solid oils | margarin |
| 1. Sugar-sweetened beverages | soft drink |
| 1. Juice | fruit juice |
| 1. Nuts and seeds | NA |
| 1. Processed meat | NA |

**Supplementary Table 2:** Expected calorie needs for guests used to adjust for the presence of guests during the 24-hour recall period

| **Age group** | **Male** | **Female** |
| --- | --- | --- |
| 0-5 | 1,030 | 1,030 |
| 6-12 | 2,267 | 2,067 |
| 13-18 | 2,900 | 2,200 |
| 19-25 | 2,900 | 2,200 |
| 26-35 | 2,900 | 2,200 |
| 36-45 | 2,900 | 2,200 |
| 46-64 | 2,410 | 1,955 |
| 65+ | 2,300 | 1,900 |

**Supplementary Table 3:** Median posterior estimate (“Estimate” column) along with 95% posterior credible intervals (“Lower bound” and “Upper bound” columns) quantifying the uncertainty in the estimation of the model parameters. Estimates are rounded to two digits. The table first displays *pi_1, pi_2, pi_3, and pi_4*, which are the population dietary pattern prevalences for each of the four dietary patterns, in order. Next, the table displays the food group consumption level probabilities for each of the dietary patterns. These parameters are displayed in order of *theta_j_k_r*, where *j* indexes the food groups and ranges from 1 to 13, *k* indexes the dietary pattern and ranges from 1 to 4, and *r* indexes the consumption levels and ranges from 1 (“Zero”) to 4 (“High”).


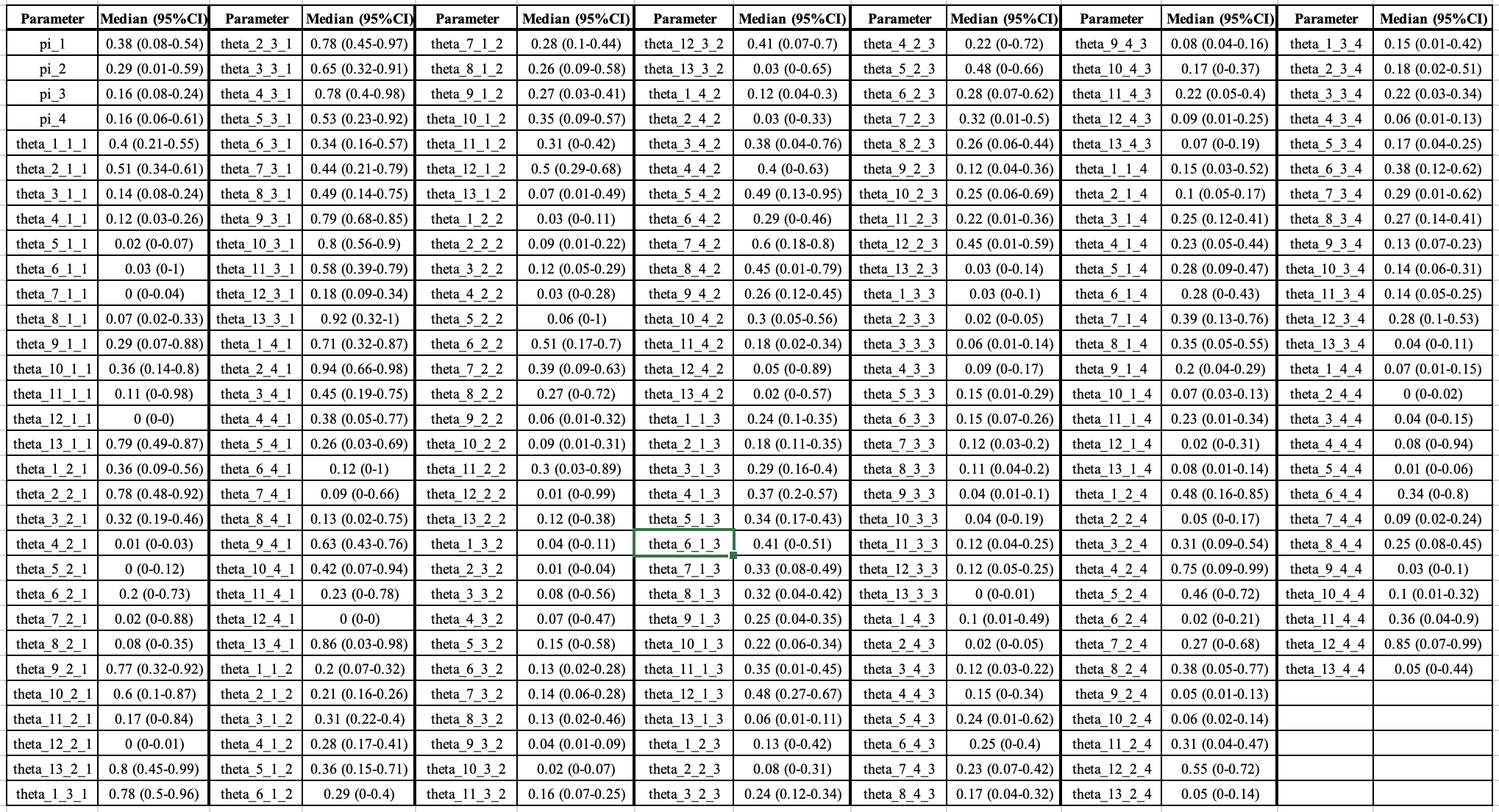


**Supplementary Figure 1:** Dendrogram of WOLCA clustering process


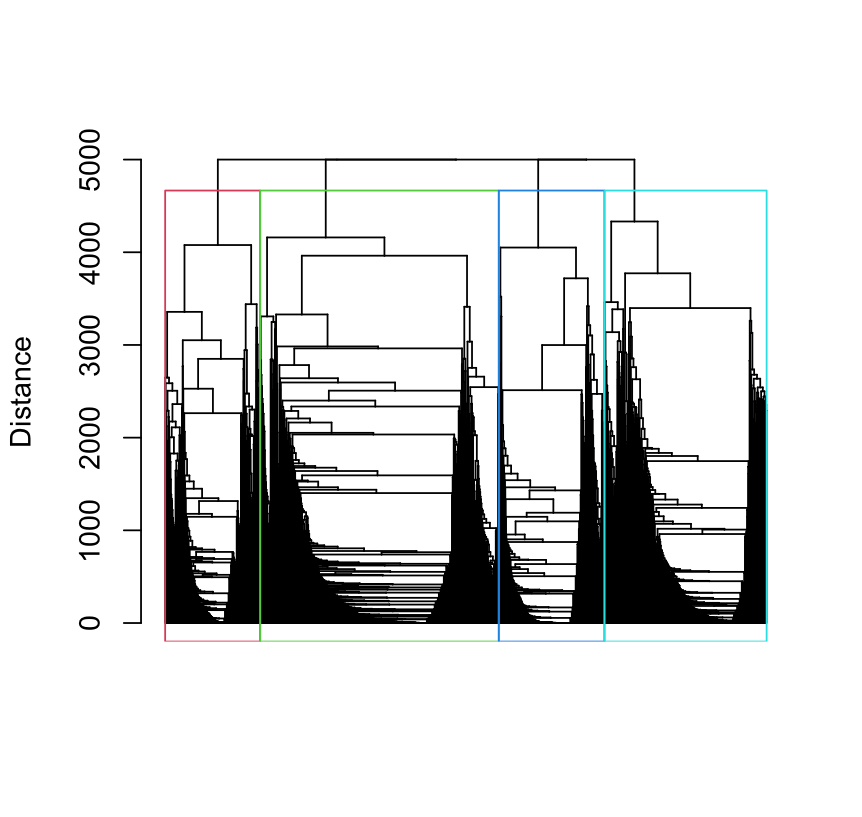


**Supplementary Figure 2:** Trace plot of WOLCA Markov chain Monte Carlo iterations for three different set seeds


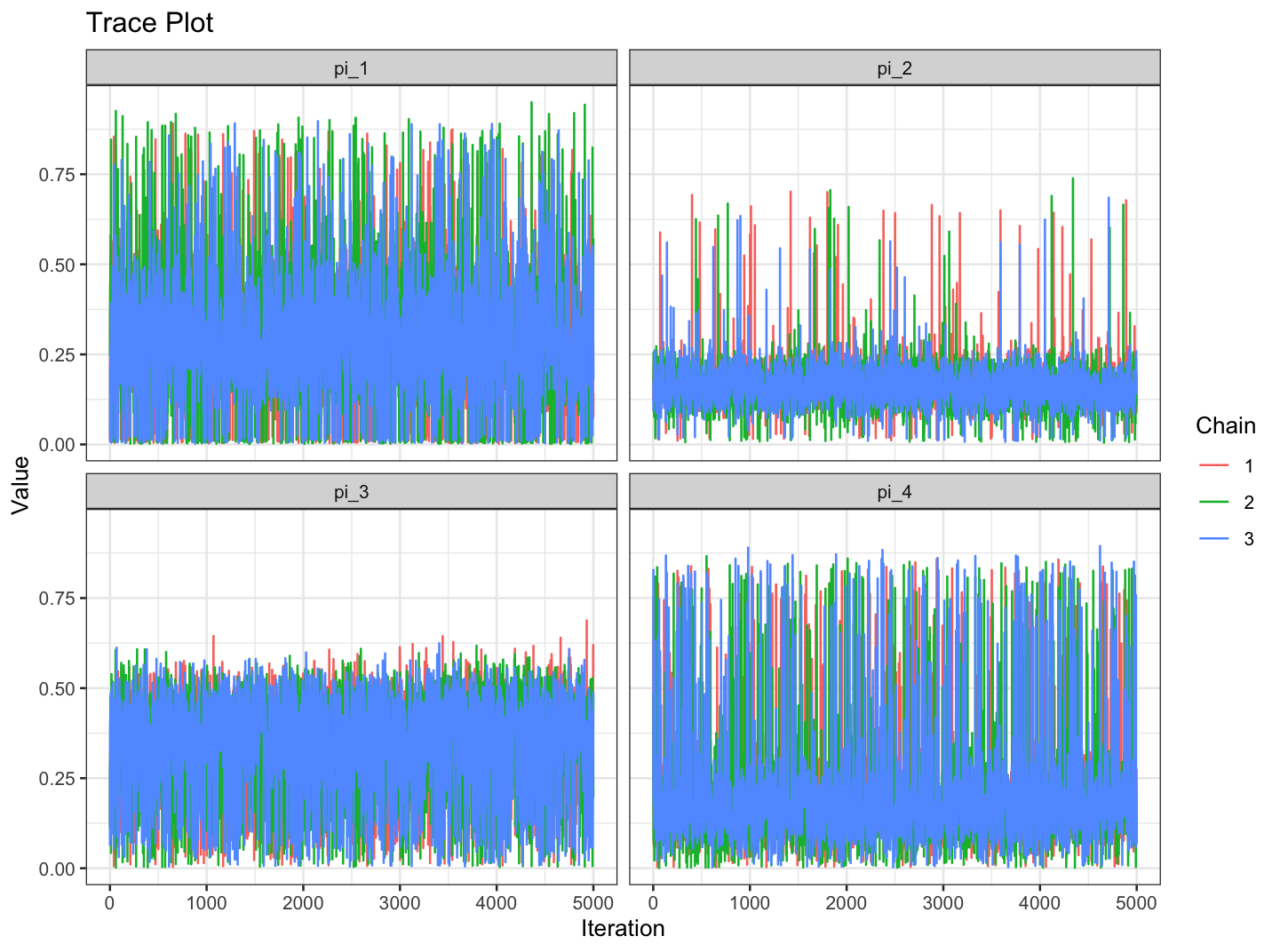


**Supplementary Figure 3:** ACF plot of WOLCA Markov chain Monte Carlo iterations


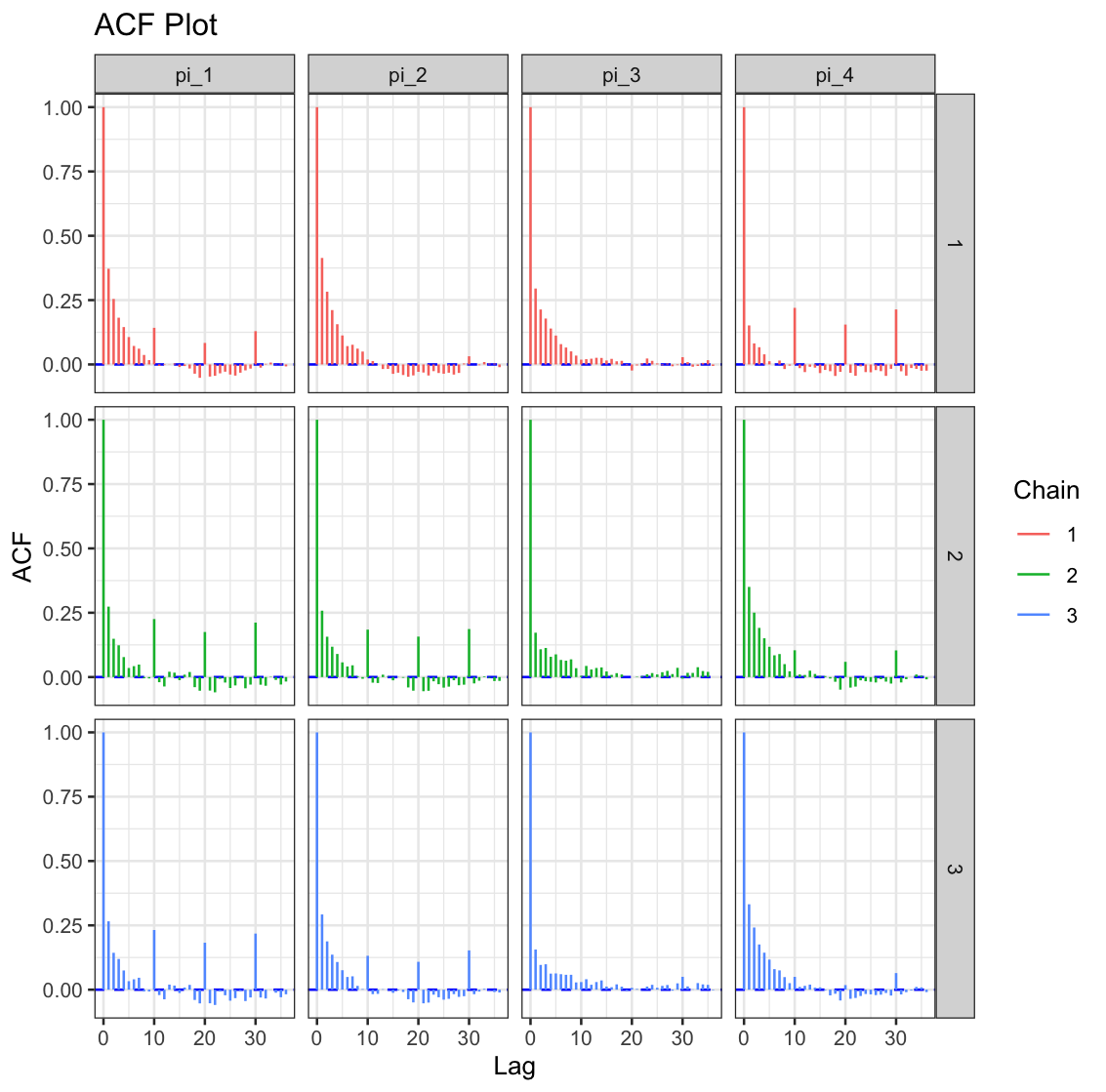


**Supplementary Figure 4:** percent of the population estimated to be included in each dietary pattern

*
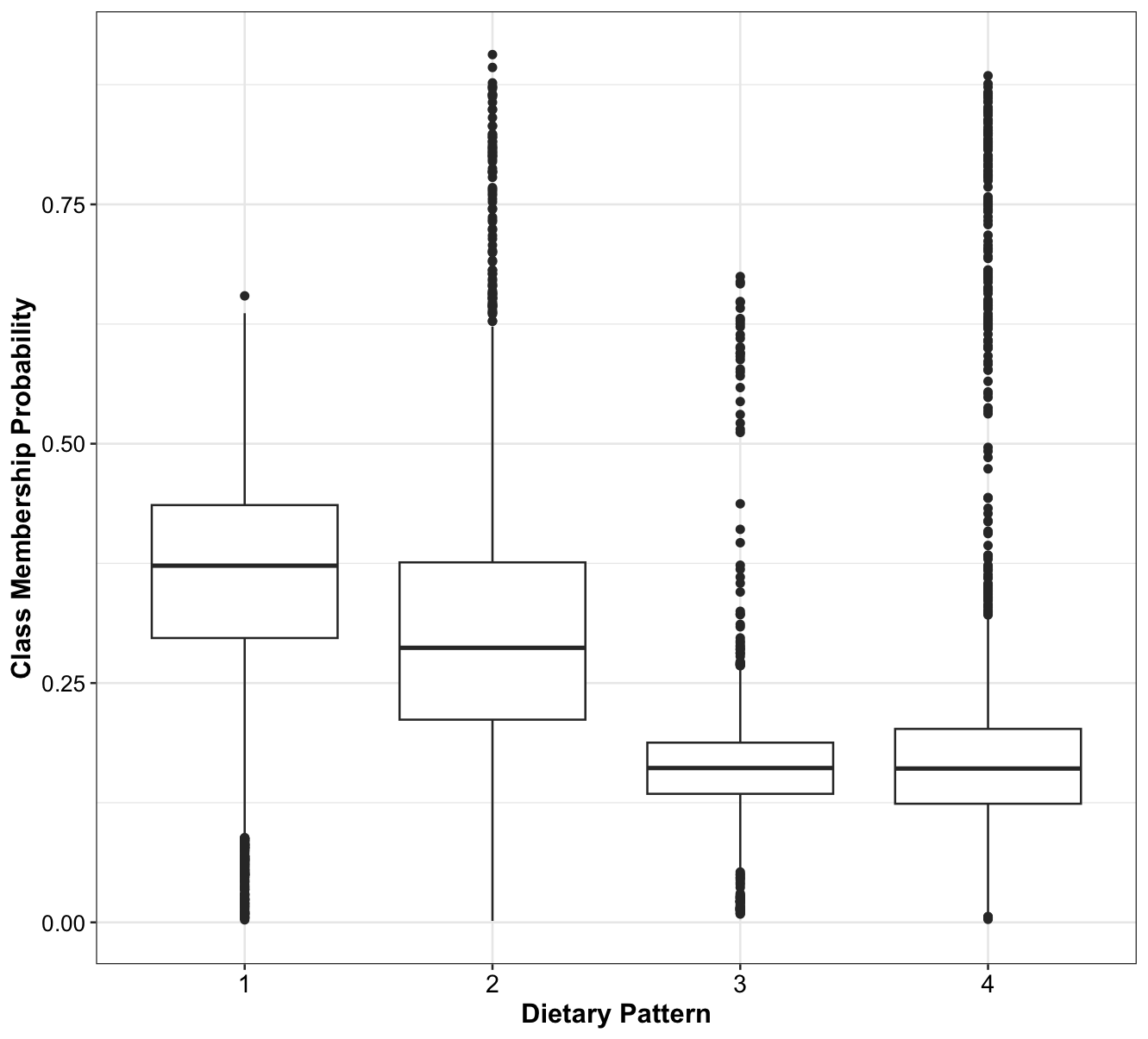
*
